# Market analysis of vitamin C-containing dietary supplements in Germany and the USA: Consumer information and risks and benefits

**DOI:** 10.1007/s00210-025-04248-y

**Published:** 2025-05-17

**Authors:** Jasmin Decke, Roland Seifert

**Affiliations:** Institute of Pharmacology, Hannover Medical School, Carl-Neuberg-Str. 1, 30625 Hannover, Germany

**Keywords:** Vitamin C, Dietary supplements, Consumer safety, Common cold, Kidney stones

## Abstract

The common cold and other flu-like illnesses are the leading cause of absence from work for adults and absence from school for children. Especially during the winter people look for an easily available and reliable way of preventing colds. Many studies have been performed on the effects of vitamin C on the common cold, and some studies have been conducted on the potential link between high doses of vitamin C and adverse effects such as kidney stones. This analysis aims to gather the available information on both beneficial and adverse effects and compare these findings to the current market of vitamin C-containing supplements. To get an accurate picture, this analysis was conducted to examine the market of vitamin C supplements further and inspect the labeling, dose, cost, and advertisement of 106 selected preparations sold by online stores in Germany and the USA. The goal was to determine what benefits and risks consumers can expect when taking the available products and if they are sufficiently informed by manufacturers. The mean daily therapy cost of vitamin C across all products was $ 0.37 for a mean daily dose of 613.9 mg. The recommended daily dose of vitamin C for adults is 90 to 110 mg and 100% bioavailability is reached from doses of 100 to 400 mg. Overall safety concerns were present among products from both Germany and the USA. For instance, more than three quarters of products exceed the recommended daily dose for vitamin C-containing dietary supplements and nearly 40% of all preparations transgress a potential upper safety level. Additionally, there were some issues with product labeling, as 12 to 14% of products from Germany and 5 to 20% of products from the USA were missing warnings that are legally required or recommended. Monopreparations were generally more popular than combination preparations. However, more than one-third of all products contained other active ingredients besides vitamin C which might increase the risk of overdoses especially if paired with other dietary supplements or multivitamins. While 80% of all products advertise vitamin C to boost the immune system, the benefits in regard to the common cold are slim, as regular vitamin C supplementation fails to lower the incidence of colds among the healthy general public. Adverse effects of even high doses of vitamin C are rare but caution is advised for people with limited kidney function or patients who have previously dealt with kidney stones. The mean daily doses of the products in this analysis will likely not lead to adverse effects among the healthy general public but might be unsafe for people who have experienced kidney stones before. In conclusion, the daily doses taken in the form of vitamin C supplements are often high, and consumers are not informed properly.

**Supplementary Information:**

The online version contains supplementary material available at 10.1007/s00210-025-04248-y.

## Introduction

**Fig. 1:**
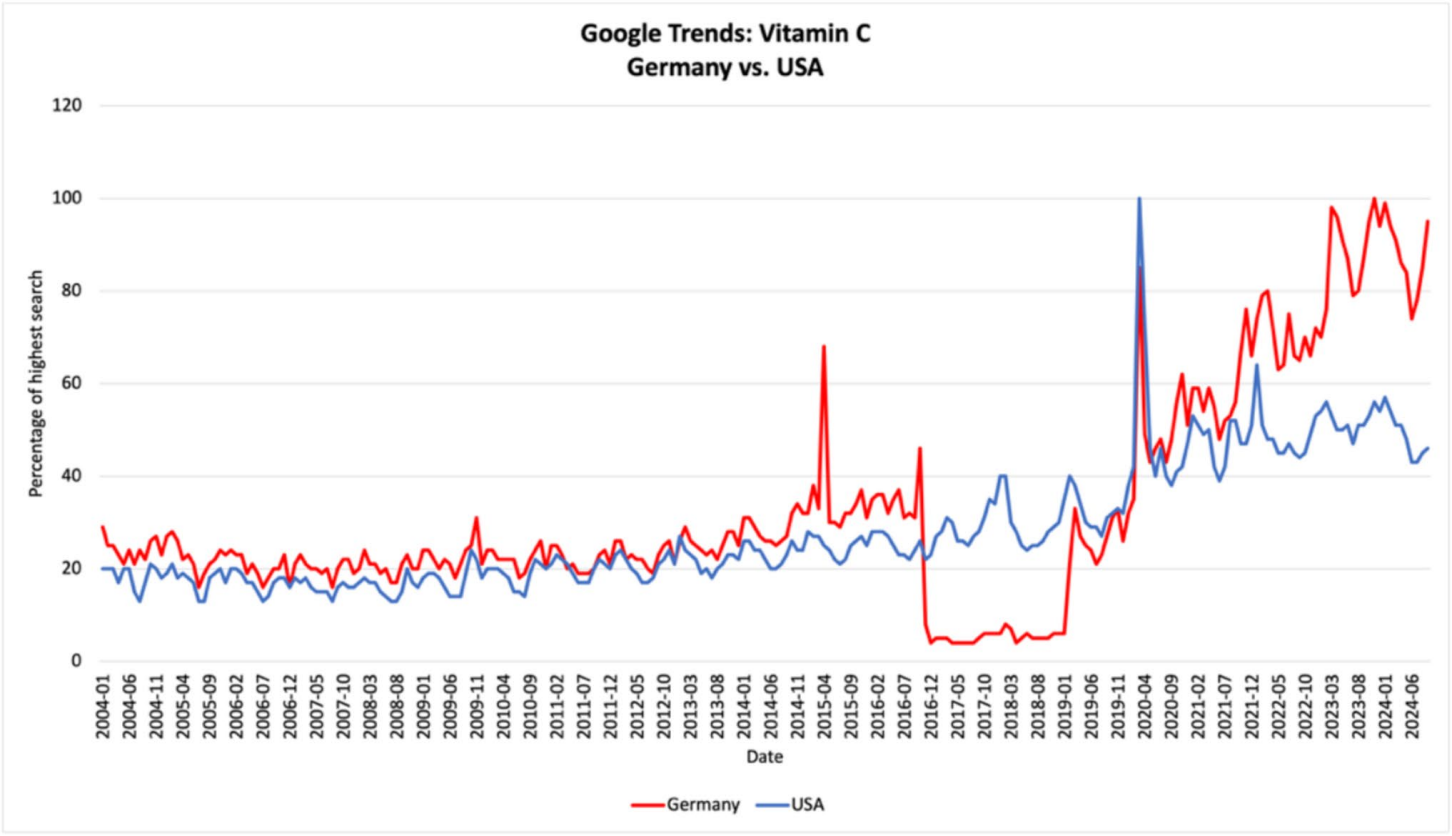
Google Trends for the search term “Vitamin C” in Germany vs. the USA from 2004 to 2024 as a trend chart via https://trends.google.de/trends/explore?date=all&geo=DE&q=vitamin%20c&hl=de and https://trends.google.de/trends/explore?date=all&geo=US&q=vitamin%20c&hl=de (retrieval on September 10, 2024), with red showing the time trend in Germany and blue showing the time trend in the USA. The number is given as a percentage of the month with the highest search respectively, which was 2023–11 for Germany and 2020–03 for the USA

The popularity of dietary supplements has seen a massive increase in the last few years with one in three people in Germany (Frankfurter Allgemeine Zeitung (FAZ), 2023) and approximately 57.6% of US-Americans (National Center for Health Statistics 2021) taking some sort of supplement on a regular basis. The global market of dietary supplements has also seen its biggest year in 2023 being valued at $ 177.50 billion (Grand View Research 2024). Among all dietary supplements, vitamin C is consistently one of the most popular ones. In Germany, vitamin C is the third most popular vitamin (32%) (Frankfurter Allgemeine Zeitung (FAZ), 2023) and in the USA, vitamin C came in at the sixth highest place of all dietary supplements (41.9%) (ConsumerLab., 2021). The number of searches of the term “Vitamin C” on Google Trends also shows a steady increase over the last few years (Fig. 1). The interest in vitamin C has grown significantly over the last 5 years with the highest interest being reported in November 2023 in Germany and March 2020 in the USA.

**Fig. 2:**
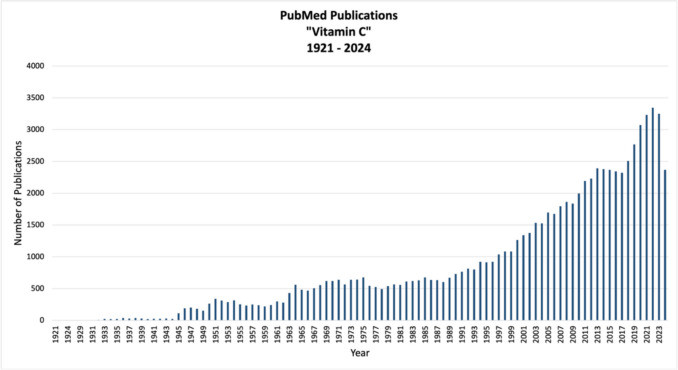
Number of publications containing the search term “Vitamin C” on PubMed as of September 10, 2024, on https://pubmed.ncbi.nlm.nih.gov/?term=vitamin+c&timeline=expanded as a bar chart

Notably, the demand of vitamin C rises during winter and cold season, as it is a common belief that vitamin C possesses the ability to prevent colds and other flu-like illnesses. Since respiratory diseases are the most common reason for absence from work in Germany with 20.6% of all absences (Statista, Radtke 2023) finding a readily available solution such as high doses of vitamin C would be of interest for employers and employees alike. The scientific interest in vitamin C as a way to potentially prevent colds (as well as other illnesses like heart diseases or cancer) has grown significantly over the past few years (Fig. 2).

Dietary supplements are easily accessible, and most pharmacies and drugstores offer a variety of preparations over the counter. It is important to note, that while supplements in the form of pills, tablets, or capsules might look like, and be promoted similarly to, pharmaceuticals, they are regulated by *food law* and are overseen by the European Food Safety Authority (EFSA) in Germany and the U.S. Food and Drug Administration (FDA) in the USA, respectively (Djaoudene et al. 2023). This analysis examines the specific regulations on dietary supplements and how they differ from regulations on drugs. Furthermore the analysis of vitamin C-containing products aims to determine how many preparations actually comply with these regulations in reality because a lack of compliance could result in adverse effects for the consumer.

Vitamin C, also known as ascorbic acid, is water soluble and therefore only possesses limited storage capacities in the human body. Since humans cannot synthesize vitamin C themselves, the daily requirement has to be met through diet (Carr and Maggini 2017). The vitamin C content of many foods, primarily fresh fruits and vegetables, is high enough to meet and even exceed the daily requirements among the German population (Deutsche Gesellschaft für Ernährung, 2015).

Vitamin C plays an important part in many vital functions and primarily acts as an antioxidant. It plays a key part in the immune system by catching free radical oxygen species (ROS) that are produced by immune cells during an immune response. High doses however show a pro-oxidant effect, which is why the plasma concentration of vitamin C is strictly controlled by the kidneys (Doseděl et al. 2021). Apart from this, vitamin C is a co-factor for the synthesis of collagen, carnitine, and certain hormones like adrenaline and noradrenaline (Doseděl et al. 2021). By affecting gene transcription, vitamin C also plays a role in epigenetic processes (Doseděl et al. 2021). Lastly, vitamin C is needed for iron absorption as it reduces ferric iron in the gastrointestinal tract and therefore directly influences how much iron is absorbed through diet (Doseděl et al. 2021).

Hypervitaminosis of vitamin C can lead to osmotic diarrhea and polyuria (Doseděl et al. 2021). Hypovitaminosis or vitamin C deficiency is rare in developed countries (Deutsche Gesellschaft für Ernährung, 2015). It can, however, lead to scurvy, a potentially life-threatening condition. Symptoms of scurvy include muscle and tissue weakness, impaired wound healing, bleeding gums (Doseděl et al. 2021), as well as an increased susceptibility to infections (Carr and Maggini 2017).

The daily value for vitamin C that is needed for optimal biological function is listed as 90 mg according to the FDA and 95 mg for women and 110 mg for men, according to the Bundesinstitut für Risikobewertung (BfR) (Table 1). This value can increase for certain groups, in which case the daily intake should be adjusted. It includes an additional 35 mg vitamin C per day for smokers, an additional 10 mg per day for pregnant women and an additional 30 mg per day for nursing women (Bundesinstitut für Risikobewertung (BfR), 2021). Furthermore, the plasma concentration of vitamin C may drop for patients with hematological neoplasias, as well as senior citizens (Doseděl et al. 2021).

**Table 1:** Recommended daily value (DV) and tolerable upper intake levels (UL) for vitamin C for adults across different sources from Germany and the USA

Source	Recommendation [in mg]
Nutrient Reference Value EU (Bundesinstitut für Risikobewertung (BfR), 2021)	80
Daily value (FDA (a), accessed September 17, 2024)	90
Daily value — women (Bundesinstitut für Risikobewertung (BfR), 2021)	95
Daily value — men (Bundesinstitut für Risikobewertung (BfR), 2021)	110
Recommendation for supplements (Bundesinstitut für Risikobewertung (BfR), 2021)	250
UL (EFSA 2004)	1000
UL (NIH, accessed March 16, 2024)	2000

When taking a vitamin C-containing dietary supplement, the BfR recommends a dose no higher than 250 mg. As of June 2024, according to the EFSA, a tolerable upper intake level for vitamin C cannot be derived due to inadequate data (EFSA 2024). The EFSA had, however, previously stated that people consuming a daily dose of up to 1000 mg should be safe from experiencing gastrointestinal issues (EFSA, 2004). This statement is considered a proposed UL for this analysis. Another source directly states 2000 mg as a UL (NIH), as seen in Table 1. Both the recommendations for supplements, as well as the UL’s, concern the *additional* vitamin C dosage on top of the daily value that is ingested through diet.

This analysis seeks to collect data on the current vitamin C market regarding daily dose and information on taking the product, including warnings and contraindications. The goal is to find out how well-informed consumers are about the products they are taking. The study also aims to compare the findings of the product analysis to scientific data on the effects of regular vitamin C supplementation in order to perform an assessment of potential risks and benefits.

## Methods

To get an accurate overview of vitamin C containing dietary supplements, 106 representative preparations sold by online stores in Germany (*n* = 66) and the USA (*n* = 40) were analyzed.

The product information was retrieved from the following online stores: *Germany* (retrieval from 12/2023 to 03/2024). https://www.shop-apotheke.com; https://www.docmorris.de; https://www.medpex.de/; https://www.dm.de; https://www.rossmann.de/de/. *USA* (retrieval from 04//2024 to 05/2024); https://www.walgreens.com.

**Fig. 3:**
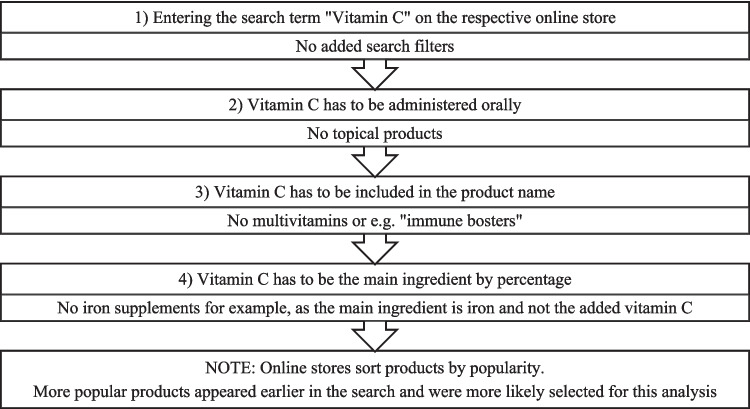
Methodical approach to finding and selecting vitamin C-containing dietary supplements. The text in the top box shows criteria that the products had to meet in order to be chosen, whereas the bottom text shows exclusion criteria

The preparations were selected based on a number of criteria (Fig. 3).

Online stores sort products by popularity; the algorithm used for this sorting is not transparent for the authors. It is, however, a reasonable assumption that the more highly recommended products are bought more frequently by consumers, which makes them of particular interest for this analysis.

Once the preparations were selected, data on them was collected in table form. Since this analysis aims to find out how well-informed consumers are when they purchase a product, only information available online was considered. Any data that was given by the online store via the product description, a picture of the product packaging or a linked leaflet was counted. If a leaflet existed but was not linked, the information was not considered in this analysis. The table collected information on the product labeling, whether it was listed as a drug or a supplement and whether a leaflet was included. Furthermore, information on monopreparations, additives, dosage form, daily therapy cost, daily dose, warnings, and advertisements was gathered.

## Results and discussion

### Drug vs. supplement

Whether a preparation is sold as a drug or a supplement has drastic effects on the legal regulations, they have to comply with. The specific legal acts and regulations in both countries are portrayed in Table 2.

**Table 2:** Compilations of legal acts and regulations controlling drugs and supplements in Germany and the USA

	Germany	USA
Drugs	Arzneimittelgesetz (AMG) (Bundesministerium der Justiz, accessed October 15, 2024)	Federal Food, Drug, and Cosmetic Act (FDCA) (FDA. (c), accessed October 19, 2024)
Supplements	Lebensmittelinformations-Durchführungsverordnung (LMIVD) (Bundesamt für Justiz. (b), accessed October 19, 2024) Lebensmittel- und Futtermittelgesetzbuch (LFGB) (Bundesamt für Justiz. (a), accessed October 19, 2024)	Dietary Supplement Health and Education Act (DSHEA) (FDA. (b), accessed October 19, 2024)

However, the difference of whether a given preparation is a drug or a supplement might not be very obvious to consumers. Of all the 106 selected preparations that meet the requirements shown in Fig. 3, 4 4 are listed as drugs, whereas 102 are listed as dietary supplements. All 4 drugs were sold by German online stores. Since the legal regulations vary not only between drugs and supplements but also between the two countries, the key differences were collected in table form and compared (Table 3).

**Fig. 4:**
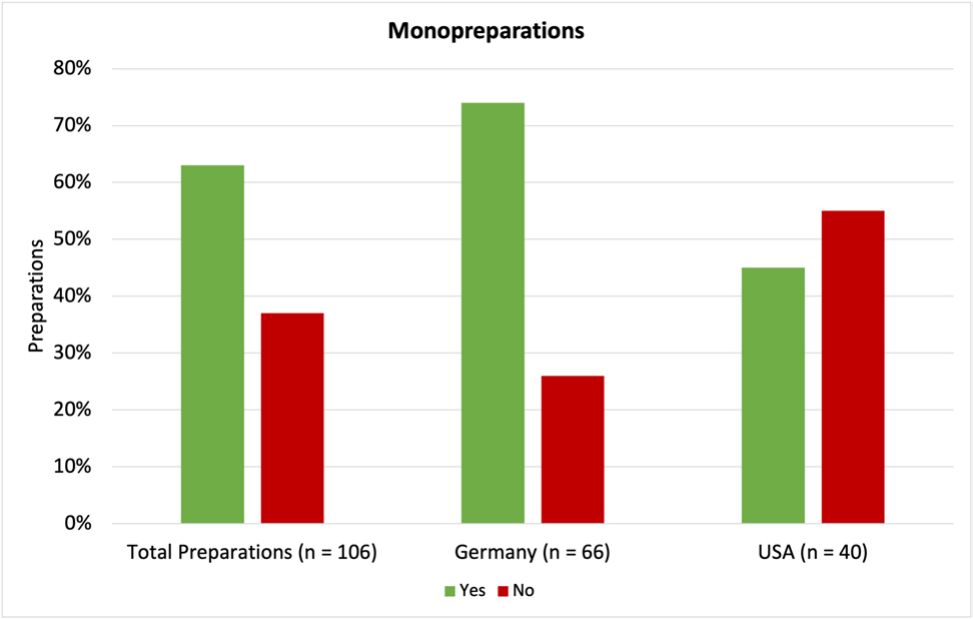
Representation of monopreparations as a bar chart, with green (“Yes”) showing monopreperations and red (“No”) showing combination preparations

**Table 3:**
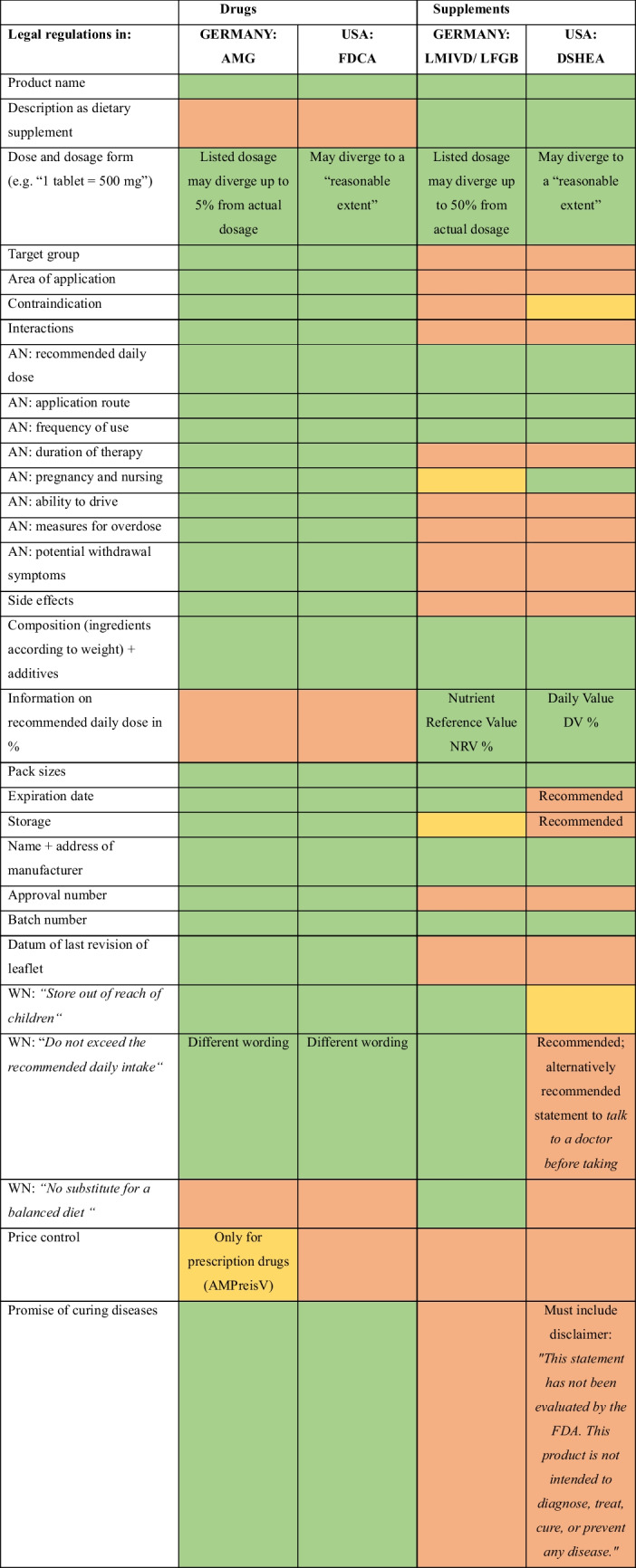
Comparison of legal regulations regarding product labeling and advertisement in Germany vs. the USA. Green indicates that the product must inform on the given topic, red indicates that there are no legally binding regulations on the topic and yellow indicates that information is only legally required for some products but not vitamin C. “AN” stands for application notice; “WN” stands for warning notice

For example, according to the German Arzneimittelgesetz (AMG), which regulates drugs, the listed dosage of an ingredient may diverge by up to 5% from the actual dosage. In contrast to that, according to the Nahrungsergänzungsmittelverordnung (NemV), which regulates dietary supplements, the listed dosage may differ by up to 50% from the actual dosage (Bundesamt für Verbraucherschutz und Lebensmittelsicherheit, accessed on September 24, 2024). The FDA states that the actual dosage may diverge from the listed dosage to a “reasonable extend,” without specifying a percentage. In general, the product labeling and marketing is far less strictly regulated for dietary supplements in comparison to drugs (see Table 3).

When taking a look at vitamin C-containing drugs, that are mainly prescribed to vitamin C-deficient patients (Tables S1 and S2), the daily dose of those drugs is quite similar to the mean daily dose of supplements at 500–1000 mg (Fig. 8). This means that consumers are taking a similar amount of the same active ingredient but legally required significantly less information. Additionally, over the counter drugs such as dietary supplements are usually taken without prior discussions with doctors or without a clear medical indication.

### Monopreparations

Of all the preparations included in this analysis, 63% were monopreparations, and 37% were combination preparations (Fig. 4). Other combination preparations such as multivitamins may also be popular, but they were not considered, because they did not include vitamin C in the product name. The results differ quite a bit between Germany and the USA. Among all products from Germany, 74% (49 preparations) were monopreparations, and 26% (17 preparations) were combination preparations. Of all US-American preparations, 45% (18 preparations) were monopreparations, and 55% (22 preparations) were combinations preparations. This shows that generally speaking, monopreparations are more popular in Germany, whereas combination preparations are slightly more popular than monopreparations in the USA.

**Fig. 5:**
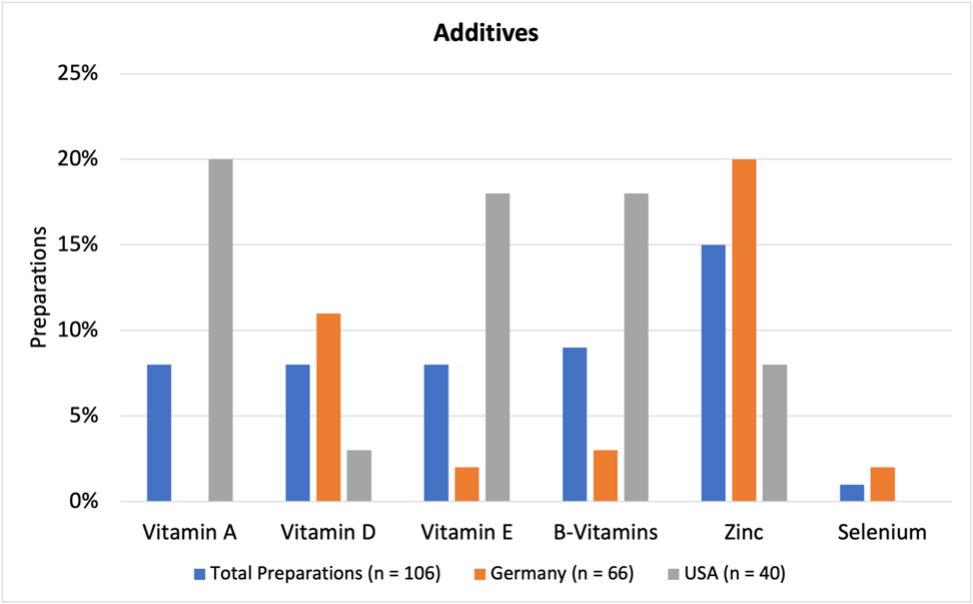
Representation of the percentage of preparations containing selected additives as a bar chart

Roughly, one-third of all preparations (39 out of 106) were combination preparations. This might be an issue, if (other) active ingredients are not labeled properly. Choosing a monopreparation is more efficient in targeting a certain issue, and keeping an overview of potential adverse effects caused by the ingredients is easier as well. Further information on other ingredients can be found in Fig. 5.

### Additives

Figure 5 shows selected additives in vitamin C-containing dietary supplements with the possibility of multiple additives being present in on preparation. Only active ingredients that were present in multiple products were selected for display. Overall, the most popular additive with 15% was zinc (16 preparations), followed by B-vitamins with 9%, and vitamins A, D, and E with 8% each. In Germany, the most popular additive was zinc, which can be found in 20% of all products (13 preparations). In descending order of frequency, other additives were vitamin D, B-vitamins, vitamin E, and selenium. For US-American preparations, the most popular additive was vitamin A being found in 20% (8 preparations). Other additives in order of frequency include vitamin E, B-vitamins, zinc, and vitamin D.

Combination preparations can pose as a health risk in two ways. Firstly, some additives may be added at a dosage that exceeds the safe upper tolerable level of the respective ingredient. Fat-soluble vitamins such as vitamin A, D, and E possess a higher risk of overdosing, as they can accumulate inside the human body. Secondly, since the additives are not the focus of the supplement, some consumers might not be aware of the fact that they are taking them at all. Even if each additive is included at a safe dose individually, if multiple dietary supplements are consumed at once, this can add up to a dosage that exceeds safe levels. Especially pairing a combination preparation with a multivitamin might pose a risk to consumers, as they cannot be expected to keep track of the total dosage of all ingredients across multiple different preparations. The general knowledge regarding safe levels of vitamins and minerals should also be taken into consideration, as it is likely that the average consumer has little knowledge about those topics. It is, therefore, important that regulations ensure safe dosage levels and clear product labeling regarding ingredients.

### Dosage forms

**Fig. 6:**
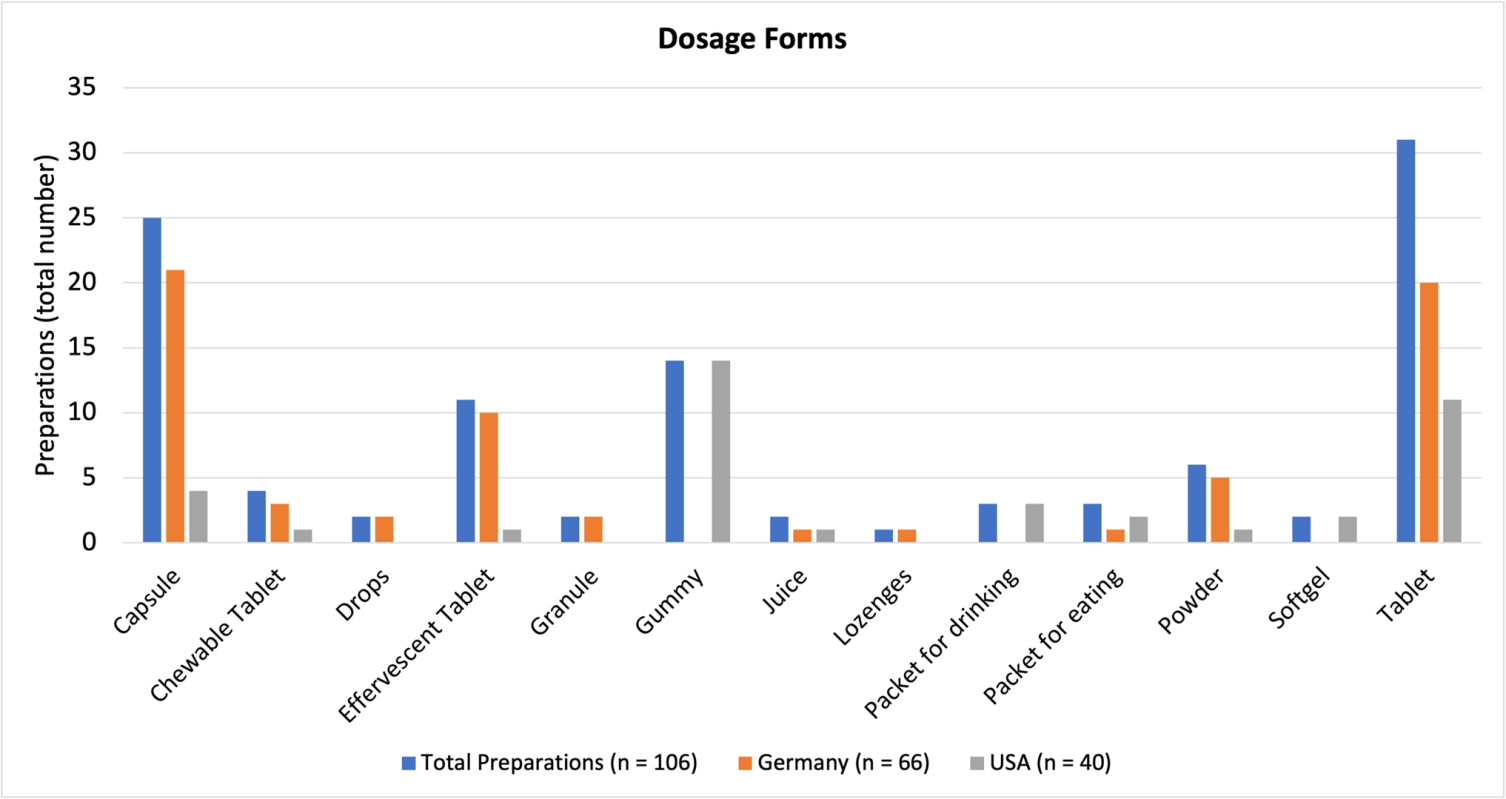
Representation of the frequency of different dosage forms as a bar chart

The most popular dosage forms among all products were tablets, capsules, and gummies (Fig. 6). The preparations from Germany (in descending order) are sold as capsules, tablets, effervescent tablets, powder, chewable tablets, drops, granules, juice, lozenges, and packets for eating. The US-American preparations (descending order) are sold as gummies, tablets, capsules, packets for drinking, packets for eating, soft gels, chewable tablets, effervescent tablets, juice, and powder. It is worth noting that not all juices are considered dietary supplements. Only when they contain added (high) doses of an ingredient are they considered a dietary supplement (as was the case with the juices included in this analysis).

The most popular dosage forms in Germany, tablets and capsules, have the advantage of being easy to store and having a long shelf life but may be difficult to swallow by some consumers. Gummies, which were the most popular dosage form in the USA, are easier to ingest but have the risk of being mistaken for candy, especially by children. Product design and labeling play a key part in making sure such a mistake does not happen. Products that contain large doses of active ingredients should be clearly labeled, and their outer appearance should vastly differ from that of candy.

#### DTC

**Fig. 7:**
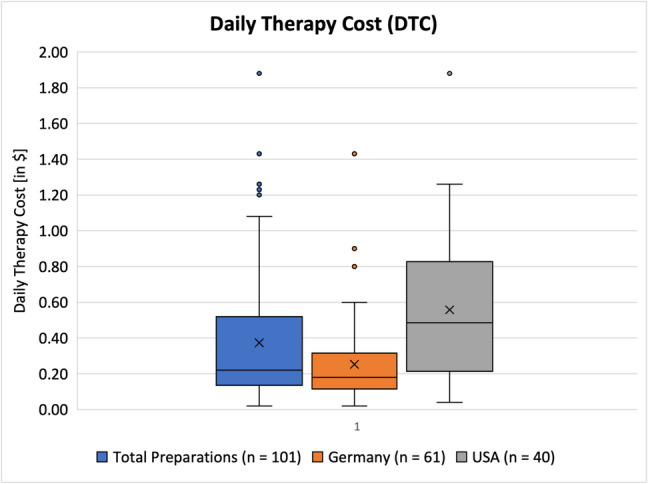
Representation of the daily therapy cost (*DTC*) as a box chart. The box correlates to the middle 50% of the data. The upper and lower whiskers mark the maximum and minimum values, respectively. The line inside of the box represents the median, while the *x* marks the mean value. Dots outside of the boxes represent outliners

Figure 7 depicts the daily therapy cost (*DTC*), according to the manufacturer. Some products recommended, e.g., “1 to 2 gummies per day” in which case the maximum amount was counted for the analysis, resulting in a higher *DTC*. The prices of the German products were originally listed in Euro and were converted into US-Dollar using the exchange rate from April 12, 2024: $1 = 0.94€. If multiple pack sizes of the same product were available, the maximum pack size was selected. Discounts offered by the online stores were not considered. Some manufacturers failed to give all the information necessary, in which case their product was excluded from the box chart. This was the case for five preparations from Germany. All US-American preparations included all necessary data on the *DTC*.

For all preparations, the mean *DTC* was $ 0.37, and the median *DTC* was $ 0.22. The minimum value in this category was $ 0.02, and the maximum value was $ 1.08. There were five outliners in total with the highest being $ 1.88. Preparations from Germany had a mean *DTC* of $ 0.25 and a median *DTC* of $ 0.18. The minimum value was $ 0.02, and the maximum was $ 0.60. There were three outliners in this category, with the highest at $ 1.43. US-American preparations had a mean *DTC* of $ 0.56 and a median *DTC* of $ 0.49. The minimum value was $ 0.04, and the maximum was $ 1.26. There was one outliner in this category at $ 1.88. When looking at the cost of long-term supplementation based on the mean *DTC*s, taking vitamin C for a 30-day month would cost $ 7.50 for German products and $ 16.80 for US products.

**Fig. 8:**
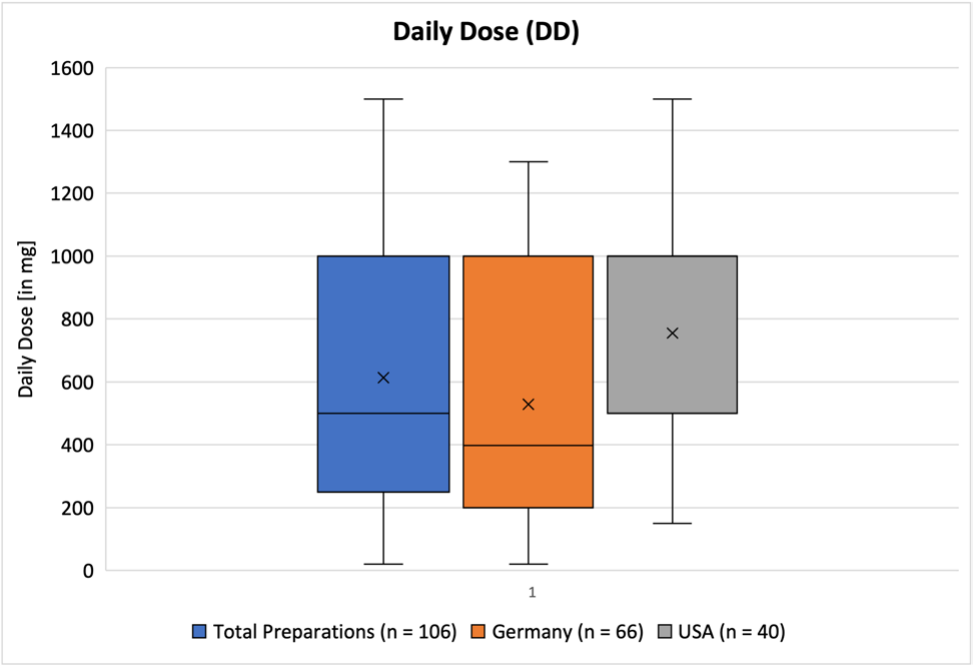
Representation of the daily dose (*DD*) as a box chart. The box correlates to the middle 50% of the data. The upper and lower whiskers mark the maximum and minimum values, respectively. The line inside the box represents the median, while the *x* marks the mean value. Dots outside of the boxes represent outliners

These numbers show that in general US-American products are twice as expensive as German ones. The mean *DD* of preparations from the USA is notably higher as well compared to Germany (Fig. 8), but while the mean *DTC* from the USA is 2.2 × that of German products, the mean *DD* of USA products is only 1.4 × more than German products. Even when weighing cost and dose, the US products are more expensive.

Furthermore, it was also noticeable that the range of prices was bigger in the USA compared to Germany as well. There are no regulations regarding the pricing of dietary supplements. In the USA, there are no regulations regarding price control at all, while in Germany only prescription drugs are subject to price control (therefore dietary supplements are not). With the mean *DTC* being $ 0.25 in Germany and $ 0.56 in the USA, respectively, it is fair to say that vitamin C-containing dietary supplements are generally affordable for the majority of consumers.

#### DD

Figure 8 shows the daily dose, according to the manufacturer. Some products recommended, e.g., “1 to 2 gummies per day” in which case the maximum amount was counted for the analysis, resulting in a higher *DD*. The daily dose was typically listed as the number in mg, as well as the percentage of the daily value. Sometimes the information given by the manufacturer was inconclusive, e.g., the listed daily dose in mg did not match the percentage of the daily value. If that was the case, the amount in mg was assumed to be the correct information.

**Fig. 9:**
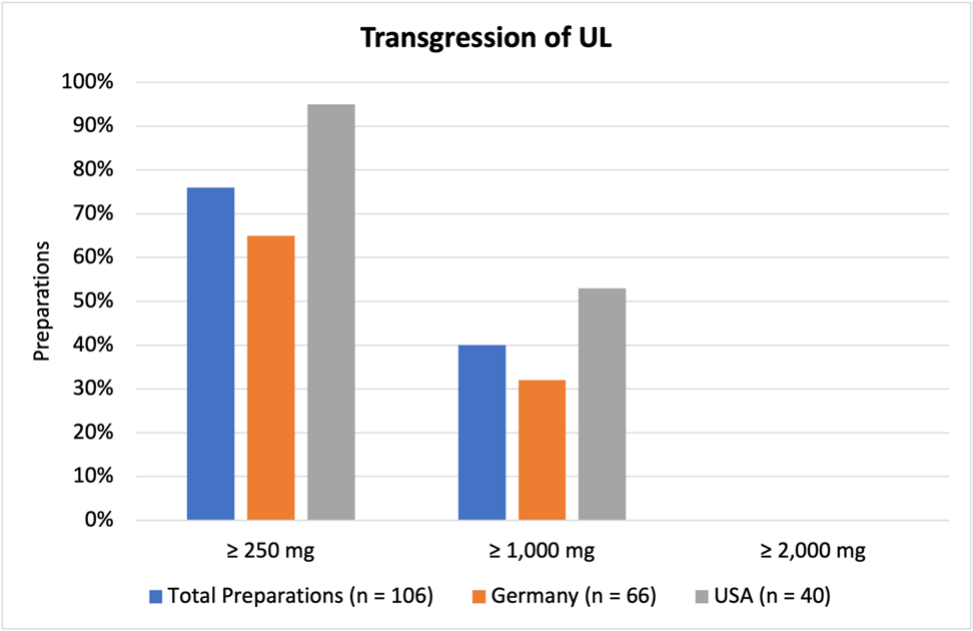
Representation of preparations that transgress the UL according to different sources as a bar chart

The mean daily dose among all preparations was 613.9 mg, and the median was 500 mg. The minimum value was 20 mg, and the maximum 1500 mg. There were no outliners in this category. For preparations from Germany, the mean *DD* was 529 mg, and the median was 397.5 mg. The minimum value was 20 mg, and the maximum value was 1300 mg. There were no outliners in this category. For all preparations from the USA, a mean *DD* of 754.1 mg and a medium of 1000 mg were found. The minimum value was 150 mg and the maximum 1500 mg. Again, there were no outliners in this category. As portrayed in Fig. 8, both the mean and median daily doses of products sold in the USA are higher than that of products sold in Germany. Figure 9 shows which daily doses transgress potential upper safety levels, and Table 5 further discusses if adverse effects are likely to occur at such doses.

Generally speaking, given a daily need of vitamin C of about 80–105 mg, the mean daily doses of the dietary supplements were about 5 to 9 times as high with 529 mg in Germany and 754.1 mg in the USA, respectively. Since vitamin C is water soluble and storage is limited, it is worth examining, if such a high dose taken at once is even absorbed by the human body. Table S2 describes the pharmacokinetics and absorption of vitamin C according to leaflets that are intended for health care providers (in German: Fachinformation). Multiple leaflets stated that for single doses of vitamin C past 1000 mg the bioavailability decreases to about 60–75%. A daily dose of 100–400 mg of vitamin C covers 100% of its bioavailability. Increasing the daily dose past that seems gratuitous, as the bioavailability is already at its maximum and cannot be increased any further (Cerullo et al. 2020). The mean daily dose among all products, as well as each individual country, does, however, exceed 400 mg, which means the dose of the preparation is likely not absorbed in full. Any excess vitamin C the body cannot absorb is excreted via urine (Cerullo et al. 2020).

### Transgression of UL

Figure 9 illustrates what percentage of preparations transgresses the UL according to different sources, as listed in Table 1. Firstly, the BfR recommends a daily dose for vitamin C-containing dietary supplements of no more than 250 mg. That amount is exceeded by 76% of all products (81 preparations), 65% of products from Germany (43 preparations), and 95% of US-American products (38 preparations). The first suggested UL of 1000 mg is passed by 40% of all products (42 preparations), 32% of products from Germany (21 preparations), and 53% of US-American products (21 preparations). No products exceed the second UL of 2000 mg vitamin C per day.

Of all products included in this analysis more than three quarters transgress the recommended daily dose for dietary supplements according to the BfR. Despite the BfR being a renowned German institution, two-thirds of German products transgress their recommendation. Almost all US-American products transgress the recommended dose of the BfR as well (as their recommendations are not well-known in the USA). It is worth noting that no US-American institution made a recommendation for supplements specifically, but instead only listed the UL of vitamin C as 2000 mg. This can explain why US-American products generally possess higher doses and more frequently pass a daily dose of 1000 mg.

Lastly, it is important to remember that recommendations for the upper levels of vitamin C were the results of expert panels that agreed on a daily dose that can be *expected* to be safe. However, those ULs are not legally binding, and no institution oversees whether a certain dose is exceeded. The obvious issue that arises is that intoxications or overdoses may occur more frequently. It is, however, difficult to give a definitive answer on what daily dose of vitamin C can be considered safe or unsafe. Therefore, it may be difficult to agree on a legally binding UL for vitamin C, as sources vary quite a lot (between 1000 and 2000 mg). Existing literature on adverse effects of vitamin C similarly does not give a conclusive answer either. The most important studies on adverse effects such as kidney stones were collected in table form (Table 5) and will be discussed under the section “Warnings on adverse effects” (Fig. 12).

Furthermore, an additional problem is that the dose listed by the manufacturer may diverge from the actual dose by high percentages, which would further increase the risk of overdoses. These high fluctuations should be less tolerated in supplements and should be closer to the tolerated divergence of the daily dose of drugs (Tables S1 and S2).

### Advertisements

**Fig. 10:**
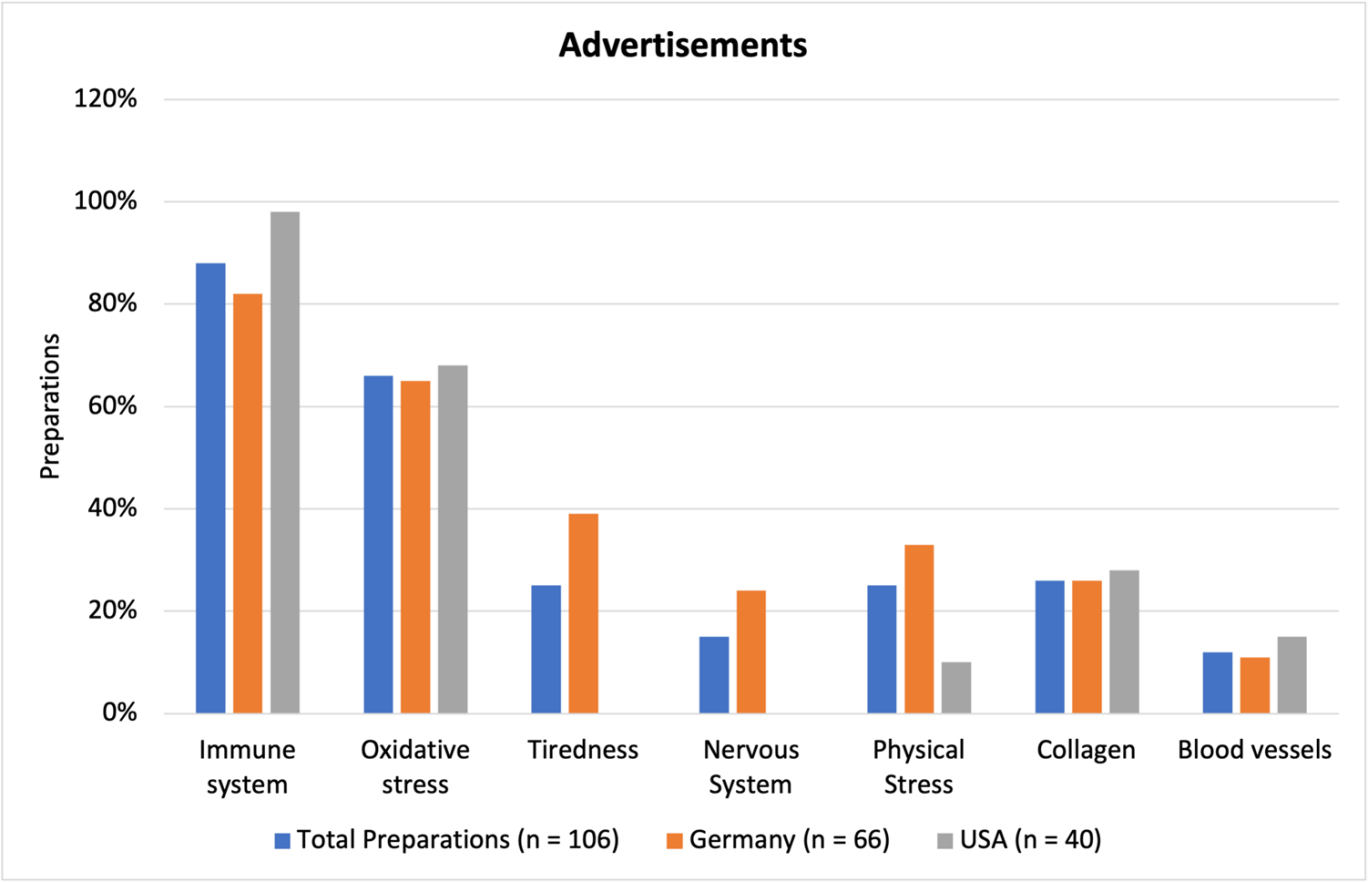
Representation of selected advertisements the preparations contain as a bar chart

Figure 10 shows what benefits the vitamin C-containing dietary supplements were advertised for. According to German laws on dietary supplements (NemV), they are not allowed to advertise that they are curing or preventing an illness or disease, but rather that they support the body’s regular functions. In the USA, manufacturers can advertise a bit more freely, but the products must contain the disclaimer “This statement has not been evaluated by the FDA. This product is not intended to diagnose, treat, cure, or prevent any disease*.*” (see Table 3).

The most popular advertisement among all products was that vitamin C can help boost the immune system or support a normal immune function, with 88% of all products (93 preparations), 82% of products from Germany (54 preparations), and 98% of US-American products (39 preparations) making such a claim. The second most common advertisement was that vitamin C helps to reduce oxidative stress, which was included in 66% of all products (70 preparations), 65% of products from Germany (43 preparations), and 68% of US-American products (27 preparations). Another advertisement was that vitamin C can counteract tiredness. This claim was exclusively found among German products and was present in 25% of all products (26 preparations), 39% of products from Germany (26 preparations), and 0% of US-American products (0 preparations). Another advertisement that vitamin C supports the nervous system was only included by German products and was present in 15% of all products and 24% among German products (16 preparations). Furthermore, some products claimed that people that are under a lot of physical stress should take a vitamin C supplement, as they have an increased daily need. Such statements were made by 25% of all products (26 preparations), 33% of products from Germany (22 preparations), and 10% of US-American products (4 preparations). Claims that vitamin C can support collagen in skin, teeth, bones, and cartilage were present in 17% of all products (28 preparations), 26% of products from Germany (17 preparations), and 28% of US-American products (11 preparations). These advertisements seem to target people that may be dealing with aging skin or osteoporosis. Sometimes mentions of collagen also included mentions of blood vessels, though sometimes they were listed as a separate benefit, which is why they are looked at as a separate advertisement. They may also target a separate group, mainly people that have had or are at a risk of cardiovascular events, such as heart attacks or strokes. The mention of supporting blood vessels was found in 12% of all products (13 preparations), 11% of products from Germany (7 preparations), and 15% of US-American products (6 preparations). Due to the way most advertisements are phrased, they could be considered truthful, as they mostly list functions that vitamin C already possesses, regardless of whether a supplement is taken or not. It is, however, questionable if a higher dose than what the body needs has any sort of positive effects or health benefits. In other words, while a deficiency of vitamin C may limit certain biological functions, it is questionable if an excess dose will increase those functions past their normal level. Currently vitamin C deficiency or risk of developing vitamin C deficiency are the only known medical indications for prescribing a vitamin C supplement (either as a drug or a dietary supplement) (Tables S1 and S2).

One common societal myth is that taking vitamin C can help prevent or cure the common cold. This idea has been popular for decades and has gained a lot of scientific interest. The most conclusive results after decades of research have been summarized in Table 4. A meta-analysis of 29 trials (Hemilä and Chalker 2013) analyzed the effects of a daily intake of at least 200 mg vitamin C per day on the incidence, duration, or severity of colds. It concluded that vitamin C generally shows no preventative effect regarding the incidence of colds among the healthy general public. However, when looking at groups that are under a lot of physical stress (e.g., athletes or military), vitamin C did show a preventative effect (Hemilä and Chalker 2013). When it comes to shortening the duration of a cold, the meta-analysis concluded a reduction of 7.7% for adults and 14.2% for children, taking at least 200 mg vitamin C per day. For larger daily doses of over 1000 mg, the duration was reduced by 8% for adults and 18.1% for children. While both sub-groups found a significant reduction, the observed effect was larger for children, as well as greater depending on a larger daily dose. Another meta-analysis (Hemilä and Chalker 2023) examined the effects on the durations of colds further and found that regular vitamin C supplementation had a significant effect on reducing the duration of severe cold symptoms but not mild symptoms. Furthermore, the severity of colds was also reduced by regular vitamin C supplementation, as the number of “days absent from school” or “days confined to house” was reduced by 15%. Since especially severe colds cause absence from work or school the effect of vitamin C supplementation on severe symptoms is of more interest. A mild but consistent effect of vitamin C shortening the duration of severe cold symptoms and the subsequent absence from work or school has been observed. Vitamin C showed no significant reduction of mild cold symptoms. Lastly, the effect of therapeutic vitamin C that is administered at the onset of cold symptoms was investigated (Hemilä et al. 2013). The meta-analysis found no consistent positive effect.

**Table 4:** Compilation of meta-analyses that examine the effects of vitamin C supplementation on the incidence, duration, and severity of the common cold

1	Citation	Hemilä et al. 2023
	Study type	Meta-analysis (15 trials)
	Participants	6258 cold episodes
	Methods	Supplementation of ≥ 1000 mg per day
	Results	Duration of common cold: • Mild symptoms: reduction by 6% [95% *KI* − 2% to 12%]—> *non-significant* • Severe symptoms: reduction by 26% [95% *KI* 15% to 35%]—> *significant* Severity of common cold: • “Days absent from school/confined to house” reduction by 15% [95% *KI* 9% to 21%]—> *significant* • “Severity Scale” reduction by 13% [95% KI: 5% to 20%]—> *significant*
2	Citation	Hemilä et al. 2013
	Study type	Meta-analysis (29 trials)
	Participants	11,306 subjects
	Methods	Supplementation of ≥ 200 mg per day
	Results	Incidence of cold during regular supplementation — general community: • *RR* = 0.97 [95% *KI* 0.94–1.00]—> *non-significant* for ≥ 200 mg per day (24 trials) • *RR* = 0.98 [95% *KI* 0.95–1.01]—> *non-significant* for ≥ 1000 mg per day (17 trials) Incidence of cold during regular supplementation — physical stress (5 trials): • *RR* = 0.48 [95% *KI* 0.35–0.64]—> *significant* Duration of common cold — adults (21 trial comparisons): • Reduction by 7.7% [95% *KI* 3.7% to 12%] for ≥ 200 mg per day • Reduction by 8.0% [95% *KI* 3.8% to 12%] for ≥ 1000 mg per day Duration of common cold — children (14 trial comparisons): • Reduction by 14.2% [95% *KI* 7.3% to 21%] for ≥ 200 mg per day • Reduction by 18.1% [95% *KI* 9% to 27%] for ≥ 1000 mg per day Severity of common cold: • Standardized mean difference of severity = 12% [95% *KI* 7% to 17%]—> *significant* (*p* = < 0.00001) for ≥ 200 mg vitamin C per day Therapeutic vitamin C after first onset of symptoms (7 study comparisons): • “No consistent effect on duration or severity of colds”

In summary, regular vitamin C supplementation does not prevent catching the common cold; it will, however, slightly lower the duration as well as relieve more severe symptoms. This is only the case for *regular* supplementation. As Fig. 1 shows, the search for vitamin C increases during the winter and cold season; therefore, it is a possible assumption that consumers only begin taking vitamin C once they start experiencing cold symptoms. This practice has shown no positive effect and will likely have no effect on the incidence, duration, or severity of a cold. The only positive effects were found when taking vitamin C long term. Considering that the average adult experiences two to four colds per year (Helios Gesundsheitsmagazin, 2024) lasting a few days each, it does not appear justified to take vitamin C all year long (or even just during winter months) just to essentially experience the same number of colds only with slightly lesser and shorter symptoms. Recently, a research paper summarized that “[…] after decades of investigations, the scientific community established that a high intake of vitamin C is useless in preventing the common cold, and therefore, a regular daily supplementation is not justified in the general population” (Cerullo et al. 2020).

Altogether, advertising vitamin C as a remedy to prevent or treat colds (short term) could be considered misleading, at least without specifying the details on duration of therapy or which sub-groups benefit the most.

### Legally required and recommended warnings

**Fig. 11:**
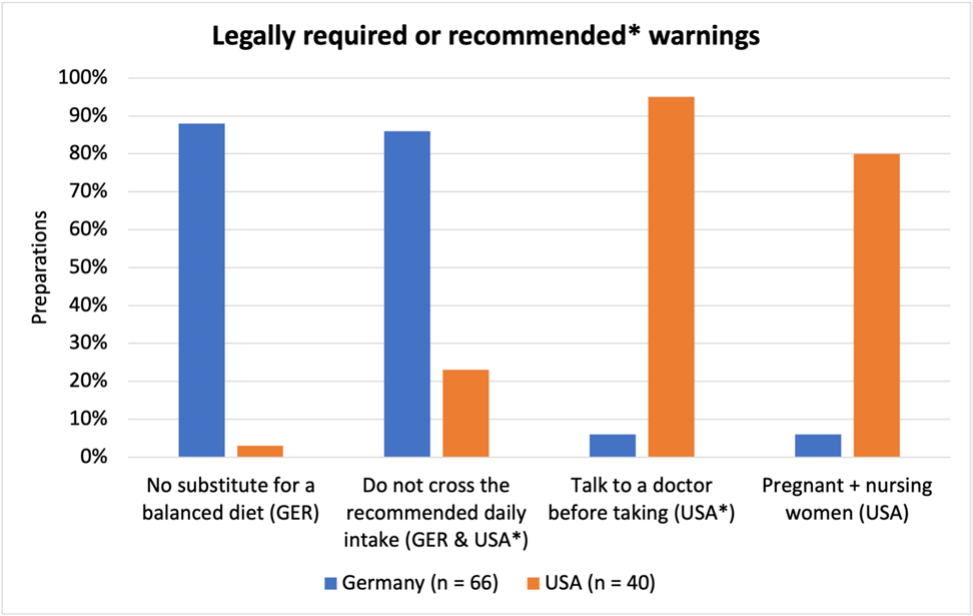
Representation of preparations containing legally required or recommended warnings as a bar chart. If a warning is legally required in Germany (GER) or the USA, the country is listed in parenthesis after the specific warning. If the country is highlighted with a star (*), the warning is not legally required but recommended. More information on legal regulations can be found in Table 3

Figure 11 shows what percentage of preparations from each country included legally required or recommended warnings. As the legal regulations differ quite a bit between the two countries, Germany and the USA were exclusively looked at as two separate groups, with no bar showing the results for all preparations. The warning *“No substitute for a balanced diet”,* which is only legally required in Germany and not in the USA, was included by 88% of products from Germany (58 preparations) and 2.5% of products from US-American products (1 preparation). Furthermore, the statement *“Do not exceed the recommended daily intake”,* which is legally required in Germany and recommended in the USA, was included by 86% of products from Germany (57 preparations) and 23% of US-American products (9 preparations). One further warning, to *“Talk to a doctor before taking”,* which is recommended in the USA, is included in 6% of products from Germany (4 preparations) and 95% of US-American products (38 preparations). The FDA suggests this warning and/or alternatively the warning to not cross the recommended daily intake. While both warnings are recommendations and not requirements, the warning to talk to a doctor seems to be favored by manufacturers. In Germany, a third warning to “store out of reach of children” is legally required but was not analyzed, as it does not primarily concern the safety of the consumer but rather of small children living in their household. Lastly, some of the products address pregnant or nursing women in their warnings, either by discouraging the use of the product for them or by recommending talking to a doctor before taking. Since vitamin C is a non-teratogenic substance, German laws do not require any mention of pregnant or nursing women, as the product is expected to be safe for use. US-American laws typically require a statement on the use during pregnancy and nursing. This warning was present for 6% of products from Germany (4 preparations) and 80% of US-American products (32 preparations).

### Warnings on adverse effects

**Fig. 12:**
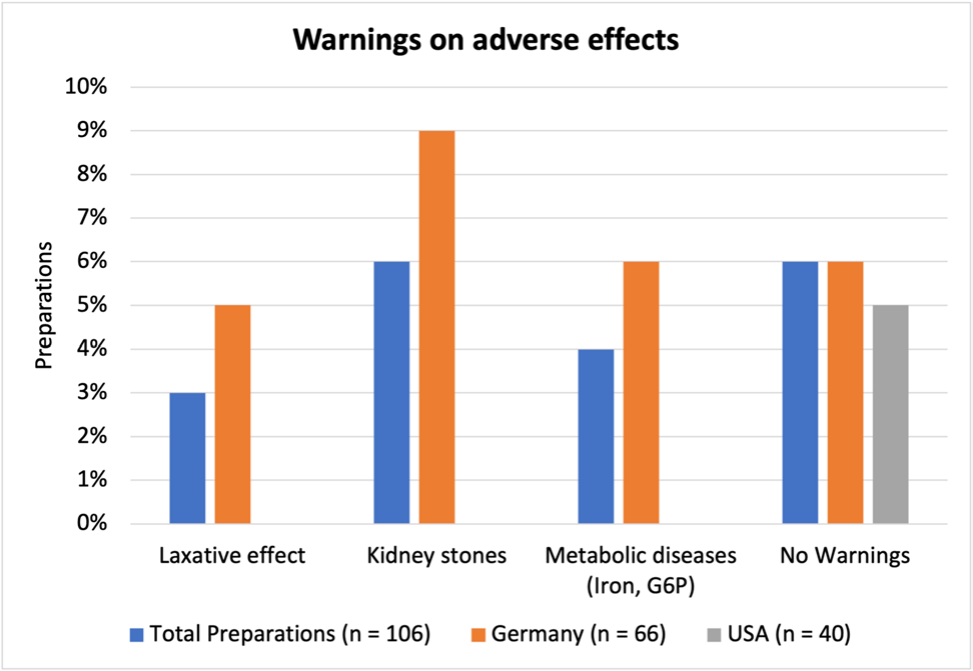
Representation of preparations containing warnings on adverse effects as a bar chart

Figure 12 shows whether warnings on potential adverse effects are included by the manufacturer. Unlike drugs, warnings on interactions or adverse effects are not required for dietary supplements (for comparison see Tables S1 and S2).

Generally speaking only German products included any warnings on adverse effects at all. The most common warning states that a high dose of vitamin C may increase the risk of developing kidney stones. Nine percent of German products or 6% of all products (6 preparations) included such a warning. A closer look into the link between vitamin C and kidney stones can be found in Table 5. Next, some products warn that a high dose of vitamin C can have a laxative effect accompanied by gastrointestinal symptoms. This warning was present in 6% of German products or 3% of all products (3 preparations). Lastly, some products contain a warning, that they should not be consumed by patients suffering from metabolic diseases, such as iron storage diseases (“iron”) or glucose-6-phosphate-dehydrogenase deficiency (“G6P”). That was the case for 6% of German products or 4% of all products (4 preparations). There were also a few products that contained no warnings at all, which included both legally required and recommended warnings (Fig. 11) as well as warnings on adverse effects (Fig. 12). This was the case for 6% of all products (6 preparations), 6% of products from Germany (4 preparations), and 5% of US-American products (2 preparations).

**Table 5:** Compilation of clinical studies that examine the link between vitamin C intake and the development of kidney stones including possible differences based on gender

1	Citation	Jiang et al. 2019
	Study type + LE	Meta-analysis (4 trials) Trials: Ferrano, 2015 (level 2b), Taylor, 2004 (level 3a), Curhan 1999 (level 3b), Curhan, 1996 (level 3b)
	Participants	131,406 men (3 trials) + 242,292 women (2 trials) = 373,698 subjects (4 trials)
	Methods	See below for the individual trials
	Results	Incidence of kidney stones in men compared to women: • *OR* = 1.62 [95% *KI* 1.09–2.42]—> *significant* (*p* = 0.02) Female subjects: • 250–499 mg: *OR* = 1.00 [95% *KI* 0.82–1.22]—> *non-significant* (*p* = 0.98) • 500–999 mg: *OR* = 1.08 [95% *KI* 0.99–1.18]—> *non-significant* (*p* = 0.09) • 1000–1499 mg: *OR* = 0.99 [95% *KI* 0.90–1.08]—> *non-significant* (*p* = 0.77) • > 1500 mg: *OR* = 0.99 [95% *KI* 0.99–1.09]—> *non-significant* (*p* = 0.88) Male subjects: • 250–499 mg: *OR* = 1.14 [95% KI: 1.00–1.28]—> *significant* (*p* = 0.04) • 500–999 mg: *OR* = 1.20 [95% KI: 0.99–1.46]—> *non-significant* (*p* = 0.06) • 1000–1499 mg: *OR* = 1.12 [95% KI: 1.11–1.13]—> *significant* (*p* < 0.00001) • > 1,500 mg: *OR* = 1.28 [95% KI: 1.00–1.63]—> *non-significant* (p = 0.05)
2	Citation	Ferraro et al. 2016
	Study type + LE	Prospective cohort study (level 2b)
	Participants	40,536 men + 156,735 women = 197,271 subjects
	Methods	Men and women were divided into 4 groups each; results were compared to placebo group (90 mg); follow-up: mean 11.3–11.7 years
	Results	Female subjects: • 90–249 mg: *RR* = 1.12 [95% *KI* 1.02–1.23]—> *significant* • 250–499 mg: *RR* = 1.09 [95% *KI* 0.97–1.24]—> *non-significant* • 500–999 mg: *RR* = 1.08 [95% *KI* 0.98–1.19]—> *non-significant* • ≥ 1000 mg: *RR* = 0.99 [95% *KI* 0.90–1.09]—> *non-significant* Male subjects: • 90–249 mg: *RR* = 1.19 [95% *KI* 0.99–1.46]—> *non-significant* • 250–499 mg: *RR* = 1.15 [95% *KI* 0.93–1.42]—> *non-significant* • 500–999 mg: *RR* = 1.29 [95% *KI* 1.04–1.60]—> *significant* • ≥ 1000 mg: *RR* = 1.43 [95% *KI* 1.15–1.79]—> *significant*
3	Citation	Massey et al. 2005
	Study type + LE	RCT (level 1b)
	Participants	27 men + 21 women = 48 subjects
	Methods	2 groups: 29 stone formers (SF) und 19 age-and-gender-matched non-stone formers (NSF); 2 phases of 6 days each: phase 1: 2000 mg daily vitamin C vs. phase 2: no vitamin C (crossover design). Measuring of urine oxalate
	Results	Effect of daily 2000 mg Vitamin C: for 40% of subjects (19 von 48) urine oxalate changed by ≥ 10% = so-called “responders “ Those were 12/29 SF and 7/19 NSF Age was a significant factor; Gender was not a significant factor
4	Citation	Taylor et al. 2004
	Study type + LE	Prospective cohort study (level 3a)
	Participants	45,619 men
	Methods	Subjects take daily ≥ 1000 mg of vitamin C; results were compared to placebo group (90 mg); follow-up: 14 years
	Results	Comparison of test group vs. placebo (90 mg): ≥ 1000 mg: *RR* = 1.41 [95% *KI* 1.11–1.80; *p* = 0.01]—> significant
5	Citation	Traxer et al. 2003
	Study type + LE	RCT (level 1b)
	Participants	24 n.d
	Methods	2 groups: “normal subjects “ and “stone formers “ Daily dose of 0 mg (placebo) or 2000 mg vitamin C in 2 phases of 6 days each; On the last 2 days of phase 2 measuring of urine pH and urine oxalate
	Results	Daily dose of ≥ 2000 mg vitamin C led to no significant change of urine pH in both groups Daily dose of ≥ 2000 mg vitamin C led to significant increase of urine oxalate in both groups Increased risk: normal subjects: 20% and stone formers: 33%
6	Citation	Curhan et al. 1999
	Study type + LE	Prospective cohort study (level 3b)
	Participants	85,557 women
	Methods	Women between the ages of 34 and 58 years without previous events of kidney stones; follow-up: 14 years
	Results	Multivariable *RR* for daily dose of vitamin C > 1500 mg compared to < 250 mg: *RR* = 1.06 [95% *KI* 0.69–1.64]—> *non-significant*
7	Citation	Curhan et al. 1996
	Study type + LE	Prospective cohort study (level 3b)
	Participants	45,251 men
	Methods	Men between the ages of 40 and 75years without previous events of kidney stones follow-up: 6 years
	Results	Age-adjusted *RR* for daily dose of vitamin C > 1500 mg compared to < 250 mg: *RR* = 0.78 [95% *KI* 0.54–1.11]—> *non-significant*
8	Citation	Urivetzky et al. 1992
	Study type + LE	RCT (level 1b)
	Participants	15 n.d
	Methods	Patients with previous events of kidney stones; vitamin C administered postoperatively after shock wave lithotripsy; Daily dose of 0 (placebo), 100, 500, 1000 or 2000 mg vitamin C for days 2 and 3 post-operatively; measuring of urine oxalate
	Results	Daily dose of ≥ 500 mg vitamin C led to significant increase of urine oxalate and therefore significantly increased risk for calcium oxalate kidney stones

These results show that warnings on adverse effects are typically present for less than 10% of products. Those numbers are compliant with current laws on labeling of dietary supplements which do not require such warnings. However, when looking at vitamin C containing drugs, which are usually prescribed at doses similar to the mean daily doses of dietary supplements with 500–1000 mg (Fig. 8), there is a striking difference in the number and types of warnings that are legally required (Table 3). Vitamin C-containing drugs must warn about potential adverse effects such as hypersensitivity reactions, diarrhea, or gastrointestinal problems. Yet only a total of 3 out of 106 products contain a warning about a potential laxative effect. Furthermore, drugs contain contraindications and list groups of people that should only take a product with caution or avoid taking it at all. Such a group is people with metabolic diseases such as iron absorption diseases or G6P deficiency (Tables S1 and S2). Again, only 4 out of 106 preparations managed a warning on this topic.

Lastly, the most serious warning is that taking vitamin C may increase the risk of developing kidney stones, as they can heavily impact a person’s overall health and wellbeing. For more than 30 years, there have been multiple studies conducted on the link between high doses of vitamin C and kidney stones. A compilation of the results of those studies can be found in Table 5. It appears that the results are still inconclusive, and while cohort studies have been conducted with many participants, other confounding factors may have affected the results. There does, however, appear to be a difference in risk of kidney stones between men and women, with men apparently possessing a higher risk (Jiang et al. 2019). Also, especially for patients with a history of kidney stones, daily doses of 500 mg and up could potentially increase the risk of developing them again (Urivetzky et al. 1992). While 6 out of 106 preparations mention the risk of kidney stones potentially associated with taking vitamin C, not one of them mentions gender as a factor. Similarly, leaflets do not comment on any potential gender-difference either, while they do recommend not to exceed a daily dose of vitamin C of 100–200 mg for patients who have previously suffered from kidney stones (see Table S1). Lastly, for patients who are at a higher risk of developing kidney stones, whether that is due to age, gender or a family disposition, warnings on vitamin C-containing supplements should be included more reliably. Additionally, further studies should be conducted to deliver more decisive results.

## Limitations

The market for dietary supplements is vast and keeps expanding every year. Therefore, it was not possible to examine every single vitamin C-containing preparation. The products selected for this analysis were chosen by a list of criteria to make the final selection as representative of the whole market as possible; however, only two countries and a total of six online stores were examined, limiting the representativity for other countries and other stores. Performing a larger analysis that includes products from multiple countries would lead to results that are even more representative of the vitamin C market. Additionally, information provided by manufacturers was limited or contradictory in some cases, and as only information given by the online stores were looked at, the authors did not have access to information that comes with the actual product, e.g., in the form of leaflets that are included in the package but were not linked by the online stores. Lastly, the products themselves or their composition were not examined, as the focus of this product analysis was placed more on how informed the average consumer is based on the given product labeling. Due to this, we were not able to determine how truthful information on dosage or additives were. The information given by manufacturers was trusted to be truthful and law-abiding.

When determining if regular vitamin C supplementation is justified, many factors play into that decision. The most important factors that were looked at were price, benefits and risks. However, not every single advertisement was analyzed in detail. We only looked at the effects of vitamin C on the immune system, e.g., when dealing with the common cold. Furthermore, when examining adverse effects, only the most severe possible adverse effect was examined. Despite research studies dating back several decades, the exact effect of high doses of vitamin C on the development of kidney stones is still not thoroughly known. Further studies will hopefully give more definitive answers regarding safe doses and possible gender differences.

## Conclusions

Firstly, when comparing products from Germany and the USA, some differences can be noticed.

In Germany, monopreparations are more popular, while combination preparations are more popular in the USA. Combination preparations may increase the risk of overdosing, especially when paired with a multivitamin. In Germany, capsules and tablets were the most common dosage forms, while the USA favored gummies. Those might pose a health risk when confused with candy, which can further increase the risk of overdoses. Generally speaking products in the USA had a higher mean daily therapy cost, as well as a higher mean daily dose. As a result, more US products transgressed a potential UL. Lastly, only German products contained any (non-legally required) warnings on adverse effects. There is no notable difference between the two countries when looking at the inclusion of legally required or recommended warnings as well as advertisements. All in all, the findings of this analysis point to German products being slightly safer than US products, but a larger analysis would be needed for more conclusive results.

In general, the majority of the vitamin C-containing supplements sold by online stores appear to be safe with low risk of experiencing adverse effects. The mean daily dose among all preparations was 613.9 mg with a mean daily therapy cost of $ 0.37. The mean daily dose far exceeds the recommended daily dose of supplements (250 mg) but does not transgress a potential UL. Therefore the daily dose can be considered safe for a healthy general public. One group that should be more cautious when it comes to vitamin C is patients that have dealt with kidney stones in the past or in general deal with kidney failure. According to leaflets of vitamin C-containing drugs (Fachinformation) people who have had kidney stones in the past should consume 100–200 mg vitamin C per day, which is far less than the mean daily dose of supplements. For kidney failure a daily dose of 50–100 mg vitamin C is advised. While patients dealing with kidney failure are most likely closely monitored by doctors, patients who have only dealt with kidney stones as a one-time occurrence might not talk to doctors as frequently. Therefore, including all necessary warnings in the product labeling is crucial for consumer safety. While most (but not all) products contain the legally required or recommended warnings, more specific warnings addressing patients who have previously dealt with kidney stones would be a justified safety measure.

Next, the benefits of a regular vitamin C supplementation appear slim. When considering decades of scientific research on the effects of vitamin C on the common cold, a regular supplementation does not appear justified, as vitamin C does not lower the incidence of the common cold and only slightly lowers the duration and severity of a cold episode. Furthermore, vitamin C only shows positive effects when taken regularly. Therapeutic approaches, i.e., beginning the intake once cold symptoms occur, have shown no positive effects. Therefore, promoting vitamin C as a cold remedy for the general healthy public is misleading. The only sub-groups that might benefit from vitamin C supplementation are those under a lot of physical stress (which can lead to lower plasma levels of vitamin C).

## Supplementary information

Below is the link to the electronic supplementary material.

Supplementary file1 (DOCX 27 KB)

## Data Availability

All original data for this study are available from the authors upon reasonable request.

## Abbreviations

AMG: Arzneimittelgesetz (= Medicinal Products Act)
AMPreisV: Arzneimittelpreisverordnung (= regulation on the prices of medicines)
BfR: Bundesinstitut für Risikobewertung
DV: Daily value
DSHEA: Dietary Supplement Health and Education Act
EFSA: European Food Safety Authority
FDA: U.S. Food and Drug Administration
FDCA: Federal Food, Drug, and Cosmetic Act
LE: Level of evidence
LFGB: Lebensmittel- und Futtermittelgesetzbuch (= Food and Feed Code)
LMIVD: Lebensmittelinformations- Durchführungsverordnung (= Food Information Regulation)
NemV: Nahrungsergänzungsmittelverordnung (= Dietary Supplement Regulation)
NIH: National Institutes of Health
UL: Tolerable upper intake level
