# Supplementary material for: Market analysis of vitamin C-containing dietary supplements in Germany and the USA: Consumer information and risks and benefits
Source: Naunyn Schmiedebergs Arch Pharmacol. 2025 May 17;398(11):15807–23. doi: 10.1007/s00210-025-04248-y (PMC12552364; doi:10.1007/s00210-025-04248-y)
Supplement: Supplementary file 1 — Supplementary file1 (DOCX 27 KB) [file 210_2025_4248_MOESM1_ESM.docx]

**Market analysis of vitamin C-containing dietary supplements in Germany and the US: Consumer information and risks, and benefits**

**SUPPLEMENT**

Jasmin Decke, Roland Seifert*

Institute of Pharmacology

Hannover Medical School

Carl-Neuberg-Str. 1

D-30625 Hannover

*to whom correspondence should be addressed

[seifert.roland@mh-hannover.de](mailto:seifert.roland@mh-hannover.de)

**Table S1** Collection of information given via leaflets by vitamin C-containing drugs sold by German pharmacies, that are intended for the consumer.

|  | **Information in leaflets for consumers** |
| --- | --- |
| Daily dose of product | 500 mg^1^  1,000 mg^2,3^ |
| Area of application | Preventing vitamin C deficiency^1,2^  Treating vitamin C deficiency^1,2,3^ |
| DO NOT use | Allergies against ingredients^1,2,3^  Current or past suffering from oxalate kidney stones^1,2,3^  Iron storage diseases^1,2,3^  G6P deficiency^2,3^  Kidney failure^3^ |
| Kidney Stones | Patients with reoccurring kidney stones should not exceed a daily dose of 100 – 200 mg^2,3^ |
| Kidney Failure | Patients with terminal kidney failure (including patients undergoing dialysis) should not exceed a daily dose of 50 – 100 mg^1,2,3^ |
| Iron Storage Disease | Patients with iron storage diseases should not take^1^ or only take after talking to a doctor^2,3^ |
| G6P Deficiency | Patients with G6P deficiency should not exceed a daily dose of 4,000 mg, otherwise they risk hemolysis^1,2,3^ |
| Interactions | - Vitamin C increases iron absorption^1,2^ - Antacids, as vitamin C can increase the absorption of aluminum, which may cause symptoms of poisoning^1,2^ - Deferoxamine in combinations with vitamin C may limit heart function^1^ - ASS/Aspirin may decrease vitamin C absorption^1^ |
| Other effects | May have an effect on laboratory results^1^:   - Blood sugar - Uric acid - Creatinine - Inorganic phosphate   May have an effect on color reactions^1^ |
| Pregnancy and nursing | Do not exceed recommended dose and talk to a doctor or pharmacist before taking^2,3^  Daily need for vitamin C is increased during pregnancy^1^  Vitamin C passed over into breastmilk^1^ |
| Recommended daily dose | Preventing vitamin C deficiency: daily dose of 50 – 200 mg^2^  Treating vitamin C deficiency: daily dose of 200 – 1,000 mg^2^ |
| Duration of therapy | Depends on symptoms and laboratory results |
| Warnings for overdoses | Seek out a doctor in case of pain in the renal area or pain during urination^1,2,3^  Diarrhea or gastrointestinal problems may occur^1,2,3^ |
| Side effects | Very rare:  Hypersensitivity reactions^1,2,3^  Headaches or dizziness^1^  Nausea, vomiting, diarrhea, heartburn, stomach aches, tiredness^1^ |
| Other hints | Healthy individuals normally are not deficient^1^  Generally, individuals from central Europe are not deficient^2,3^  Increased daily need:   - Senior citizens^1,2,3^ - Smokers^1^ - Pregnant women^1^ - Infants fed with cow milk^2,3^ |
| Information as of the following date | October 2020^1^  October 2021^2^  April 2016^3^ |

**The following leaflets were examined:**

1 = Cetebe Vitamin C Retard 500

2 = Wörwag Pharma Vitamin C 1000

3 = HERMES Cevitt Zitrone (Brausetablette)

**Table S2** Collection of information given via leaflets by vitamin C-containing drugs sold by German pharmacies, that are intended for health care providers. This table only shows relevant information that goes beyond the information given in leaflets for consumers, as the information is identical and can already be found in Table S1.

|  | **Information in leaflets for health care providers** |
| --- | --- |
| Daily dose of product | 500 mg^1,3^  1,000 mg^2^ |
| DO NOT use | Hereditary diseases such as^1,2,3^   - Fructose intolerance - Galactose-glucose-malabsorption - Saccharose-isomaltase-deficiency |
| Pregnancy and nursing | Vitamin C passes the placental barrier^1,2,3^  High doses of vitamin C during pregnancy leads to a higher risk of developing scurvy for the newborn^2^ |
| Warnings for overdoses | Osmotic diarrhea and gastrointestinal symptoms occasionally occur after a single dose of vitamin C > 3,000 mg and almost always occur after a single dose of 10,000 mg |
| Other hints | Vitamin C deficiency^1,2^:   - plasma concentration of vitamin C < 6 μmol/L (0,1 mg/dl) - concentration of vitamin C in white blood cells < 280 μmol/L (5 mg/dl)   Lower limit of sufficient vitamin C plasma concentration^1,2^:   - Men: 0,5 mg/dl - Women: 0,55 mg/dl   Amongst senior citizens, men are at a higher risk of vitamin C deficiency than women^1,2^ |
| Pharmacodynamics | Vitamin C is an important part of the redox system^1,2,3^  Deficiency limits immune function^1,2,3^  Vitamin C improves iron absorption^1,2,3^  Vitamin C has an antioxidative function^1,2,3^  A protective role against potentially cancerogenic substances in the gastrointestinal tract has not been scientifically proven^1,2,3^ |
| Pharmacokinetics | Absorption^1,2,3^:  If a single dose exceeds 1,000 mg the bioavailability decreases to about 60 – 75%;  past 3,000 mg it decreases to about 40% and  past 12,000 mg it decreases to about 16%  Distribution^1,2,3^:  Maximal metabolic turnover of 40 – 50 mg/ day for a plasma concentration of 0.8 – 1.0 mg/dl  Under high doses of oral vitamin C a plasma concentration of 4.2 mg/dl may be reached temporarily and under these condition > 80% is excreted via urine  Elimination^1,2,3^:  Excretion via urine; half-life 2.9 hours  Upper limit for healthy individuals:   - Men: 1.34 ± 0,21 mg/dl - Women: 1.46 ± 0,22 mg/dl |
| Preclinical data | No subchronic or chronic toxicity  No mutagenic effect  No tumorigenic potential  No fetotoxic effect |
| Information as of the following date | April 2023^1^  October 2021^2^  February 2019^3^ |

**The following leaflets were examined:**

1 = VITAMIN C-ratiopharm retard 500 mg

2 = Wörwag Pharma Vitamin C 1000

3 = Xitix 500 mg Lutschtabletten
